# The Clinical Congress

**Published:** 1914-08-08

**Authors:** 


### THE PROFESSION OF MEDICINE.

### The Clinical Congress.

### PERSONAL IMPRESSIONS OF THE CONCLUDING STAGES.

On the last two evenings of the session of the Clinical Congress of Surgeons of North America, some exceedingly important papers were read, and some interesting personalities were revealed to the assemblage. On July 30 the feature of the evening was to have been a paper by Professor Krönig, of Freiburg, on the treatment of uterine tumours by $x$-rays and radium. To the disappointment of the audience, it was announced that the eminent Continental surgeon had been obliged to return to Europe, owing to the events which have since culminated in the war. In his absence, a New Orleans surgeon came forward and most obligingly stepped into the breach at very short notice. Having lately visited Freiburg, he was able to summarise for the Congress the views which Professor Krönig and Professor Aschoff hold upon this particular subject; and his paper was both extraordinarily interesting and remarkably well put together considering the brief notice at which it was composed. In a sense it was even more valuable than the contribution expected of Professor Krönig could have been, for it represented the views of an observer able to take a more impartial view of the work done at Freiburg than any of the actual authors of that work could be. At all events, it was made clear that the Freiburg professors have almost given up operating for uterine tumours, both innocent and malignant, with the exception of myomectomy for fibroids in women under forty, which they still practise when it is feasible.

#### Radium and Moderate Heat Treatments.

They claim, apparently, that by $x$-rays and by radium, applied as they direct, results superior to those of operation can be obtained in the treatment of these conditions. As the speaker was careful to add, these claims are not accepted at other important Continental clinics—indeed, they are combated and ridiculed more strenuously in the country of their origin than outside it. It was also made clear that the reporter does not endorse Professor Krönig's teachings—at any rate at present. While keeping an open mind on the matter, he professed himself not convinced by what he saw and heard at Freiburg, though much interested and distinctly impressed by the enthusiasm and ability of Professor Krönig.

The discussion was thus set going along "safe" and non-controversial lines, but the next speaker, Dr. J. F. Percy, of Galesburg, Illinois, diverged at once from all well-beaten tracks. He described his methods of treatment by moderate heat—a kind of slow cooking process, by which he maintains that he is able to destroy cancer cells while leaving normal tissues uninjured. The proof of the pudding is, of course, in the eating; and if Dr. Percy's results are really as good as he believes, he will have written his name indelibly on the scroll of medical fame. His presentation of his ideas, however, bristled with improbabilities and unproved assumptions, and evidently failed to impress the bulk of the Congress as scientifically sound. It is pleasing to add that a leading London institution, the Cancer Hospital, promptly offered him the chance of putting his methods into execution; and that on the following day (July 31) he operated there upon two cases of uterine cancer deemed inoperable by ordinary surgical skill.

#### Statistics and Results.

Professor Wilson's contribution to the debate was thoughtful and worthy of his reputation. He laid his finger upon a weak spot in the English literature of uterine cancer when he pointed out that there are no published statistics by any British operator of the results of Wertheim's operation, based upon a five-years' test of cure. This deficiency Professor Wilson very laudably set himself to supply, and his figures show an "absolute curability" of 10.2 per cent. This phrase means the proportion to the total cases seen of those who survive operation for five years without signs of recurrence. The figure seems at first sight a low one, but it has to be remembered that under a third of the patients are found to be operable at all—that is, the great majority are in a hopelessly advanced stage of the disease when they first come into the surgeon's hands. Compared with Professor Wertheim's statistics, and others from other non-British sources, this 10 per cent. ratio is not brilliant; but it undoubtedly represents facts, which not all statistics can be trusted to do, and we doubt whether any English operator's results, analysed as conscientiously, would disclose a better showing.

Of course, as was pointed out by a subsequent speaker (Dr. Eden), and quite justly, the mere fact that the first three papers recommended three perfectly different and fundamentally distinct methods of treating one disease is presumptive evidence that the last word has not yet been said on the subject. Professor Wilson's statistics represent surgical results which will doubtless be considerably improved as earlier diagnosis and more exact technique are secured; yet it will require a vast improvement before the present "absolute curability," now 10 per cent., can be regarded as other than disappointing. One can agree with Dr. Eden that Wertheim's operation alone cannot be expected to carry matters much further than they at present stand. Though it will probably be practised for many years yet, it will have to be assisted by other measures, possibly radiological, possibly bio-chemical, before it can provide us with a really satisfactory defence against this disease.

On the next evening, July 31, the first paper was by Professor Rodman on cancer of the breast.

Diagnosis was chiefly dealt with, and was very thoroughly gone into. Nevertheless, very little of any novelty to British surgeons was promulgated; it would seem as if surgical education in respect of this particular kind of cancer is more advanced in this country than in America. The account of the speaker's operative technique was interesting. There is a good deal to be said theoretically in favour of the mode of access which was demonstrated in lantern slides; but the operation must be made a good deal more difficult and lengthy, one would think, than that which is usually practised in this country. The final result is the same in both cases, so it seems doubtful whether Professor Rodman's technique is really much of an improvement. This paper was discussed by Mr. W. Sampson Handley, who fell into the error—made by a good many other surgeons in the previous sessions—of reading an independent paper of his own instead of debating that already read. Mr. Handley's paper chiefly consisted of a recapitulation of his researches of ten years ago. Valuable as they were, there was no need to explain them all over again to a body of surgeons most of whom must have been perfectly familiar with them already.

### A Medical Debater.

Then came a symposium on intestinal stasis, which Professor J. B. Murphy, the president, foretold would be a battle of the giants. The first speech—there were no formal papers read—was delivered by Sir Berkeley Moynihan, and was an intellectual treat of a very high order. This well-known Leeds surgeon is the happy possessor of a capacity for debate which would speedily make a great reputation in the House of Commons. He marshals his thoughts, without the aid of notes, in orderly logical development; and he clothes them in language of crystal clearness and simplicity beyond the possibility of misunderstanding. His impromptu speech might be taken down word for word by a shorthand writer, and printed as a model of correct and graceful English composition, neither too rhetorical nor too bald, neither florid nor devoid of sparkle. He uses a naturally sonorous and pleasing voice with great skill; the most perfect articulation and delivery enhancing the effect of the merits already described. He speaks without effort and almost without pause, yet in unhurried and steady regular flow. He has a playful wit and a mobile face. In a word, Sir Berkeley Moynihan is the most excellent of all the orators who addressed the Congress, and his oratory is of exactly that type which appeals most to Englishmen. The line he took with regard to intestinal stasis, or rather with regard to the claims of the school which follows Sir Arbuthnot Lane, was sane and sound. Admitting the great merits of much of that work, he was as yet unable to assent to the latest developments of it until further proofs have been submitted and stringently examined. That is to say, he was properly sceptical, yet willing to be convinced by proper evidence.

### "An Affair of Outposts."

Sir Bertrand Dawson, who spoke next, took up an attitude more or less in line with Sir Berkeley Moynihan. Coming after the latter's brilliant speech, Sir Bertrand was palpably overshadowed, though in the ordinary way his speech would certainly have been deemed above the average in excellence. Following him, Professor Bloodgood, of Baltimore, addressed the meeting. It was speedily obvious that this surgeon had nothing of much note or value to contribute to the discussion, and as this gradually impressed itself upon the audience symptoms of weariness eventually became manifest. Undeterred by the obvious indications of this, Professor Bloodgood continued at great length, and even the President's repeated appeals to him failed to induce him to conclude. It was a lamentable exhibition, and the result of his long-windedness and obstinacy was that Sir Arbuthnot Lane, whom everyone wanted to hear, was practically deprived of his opportunity. When he at last got his chance the hour was so late, the audience so fatigued, and the air in the hall so stifling, that he contented himself with a little genial chaff of Sir Berkeley Moynihan and a reiteration of belief in his position as defined in his published writings. Thus the battle of the giants turned out to be an affair of outposts only, though redeemed, as has been said, by the magnificent speech of the famous Leeds surgeon.

